# A novel mechanobiological model can predict how physiologically relevant dynamic loading causes proteoglycan loss in mechanically injured articular cartilage

**DOI:** 10.1038/s41598-018-33759-3

**Published:** 2018-10-22

**Authors:** Gustavo A. Orozco, Petri Tanska, Cristina Florea, Alan J. Grodzinsky, Rami K. Korhonen

**Affiliations:** 1 Department of Applied Physics, University of Eastern Finland, Kuopio, Finland; 2 Departments of Biological Engineering, Electrical Engineering and Computer Science and Mechanical Engineering, Massachusetts Institute of Technology, Cambridge, MA USA

## Abstract

Cartilage provides low-friction properties and plays an essential role in diarthrodial joints. A hydrated ground substance composed mainly of proteoglycans (PGs) and a fibrillar collagen network are the main constituents of cartilage. Unfortunately, traumatic joint loading can destroy this complex structure and produce lesions in tissue, leading later to changes in tissue composition and, ultimately, to post-traumatic osteoarthritis (PTOA). Consequently, the fixed charge density (FCD) of PGs may decrease near the lesion. However, the underlying mechanisms leading to these tissue changes are unknown. Here, knee cartilage disks from bovine calves were injuriously compressed, followed by a physiologically relevant dynamic compression for twelve days. FCD content at different follow-up time points was assessed using digital densitometry. A novel cartilage degeneration model was developed by implementing deviatoric and maximum shear strain, as well as fluid velocity controlled algorithms to simulate the FCD loss as a function of time. Predicted loss of FCD was quite uniform around the cartilage lesions when the degeneration algorithm was driven by the fluid velocity, while the deviatoric and shear strain driven mechanisms exhibited slightly discontinuous FCD loss around cracks. Our degeneration algorithm predictions fitted well with the FCD content measured from the experiments. The developed model could subsequently be applied for prediction of FCD depletion around different cartilage lesions and for suggesting optimal rehabilitation protocols.

## Introduction

Articular cartilage is a well-organized poroelastic tissue that covers the ends of bones in diarthrodial joints and performs as a low-friction, load-bearing surface for efficient articulation. Cartilage is composed of an extracellular matrix (ECM), which consists of a highly hydrated ground substance, predominantly composing of aggrecan proteoglycans (PGs) and their negatively charged glycosaminoglycan (GAG) chains, reinforced by a fibrillar collagen network, all synthesized by chondrocytes. The PGs control water content through osmotic pressure and GAG-GAG repulsive electrostatic interactions, straining the collagen fibrils and resisting compressive loads. Unfortunately, traumatic joint loading can disrupt this complex structure, produce lesions in cartilage^1–3^, and lead to post-traumatic osteoarthritis (PTOA).

Various experimental^4–12^ and numerical^13–19^ studies have investigated cartilage changes in knee PTOA, those are typically PG loss, increase in water content and permeability, disorganization and disruption of the collagen matrix. As a consequence of the initial cartilage lesion, the fixed charge density (FCD) (associated with GAG chains of PGs) and tissue swelling may decrease near the injury, reducing the tissue stiffness, and weakening the ability of the organized collagen network to resist tensile forces^20^. In fact, it has been suggested that **(i)** the FCD loss appears before collagen matrix damage over a short period of follow-up time^21,22^ and that **(ii)** changes in the collagen network organization are small around cartilage lesions and larger at the cartilage-bone interface^23^. Furthermore, several experimental studies have suggested that collagen content does not change in early PTOA, but rather follows other structural and compositional changes^24–26^. These findings support the analysis and prediction of FCD loss as the first degenerative sign.

Mechanisms leading to these aforementioned tissue changes are not well understood and they cannot be currently predicted^4^. Recently, computational methods have contributed to an understanding of the evolution of tissue damage after cartilage injury, mainly based on tissue deformation as a predictor^13,15,27^. However, absence of realistic lesions in the models, as well as insufficient experimental comparisons have limited the validity of the numerical predictions. Moreover, other mechanisms should also be explored, for instance, fluid flow velocity has been used to predict the bone formation patterns observed experimentally during bone generation procedures^28,29^. When mechanical loading is involved after the cartilage injury, **(i)** the initial collagen disruption could cause FCD leakage through the damaged surface due to high interstitial fluid flow velocity by assuming that particles are released from the free lesion surface through fluid expulsion^30–32^, or **(ii)** tissue loading could cause increased strains in the vicinity of the lesion, leading to a localized cell death and subsequent FCD decrease^15^.

Here we develop a mechanobiological cartilage degeneration model with a **(i)** fluid velocity, **(ii)** deviatoric strain, and **(iii)** maximum shear strain controlled algorithms, and employ this model to simulate changes in the tissue FCD around cartilage lesions when a moderate (normal), physiologically relevant dynamic loading is applied to cartilage. The results are compared to the experimentally observed FCD loss of cartilage around cracks *in vitro* in the absence of exogenous inflammatory cytokine challenge. We hypothesize that, due to the non-uniform strain distributions found earlier around cartilage lesions^30,33,34^, the strain-based algorithm in the models causes a non-uniform FCD loss. On the other hand, because fluid pressure at the inner crack surface is negligible and uniform through the crack depth, the FCD loss is hypothesized to be more uniformly distributed around lesions in the fluid velocity-controlled degeneration algorithm. Prediction of these compositional changes with the model could help to recognize lesions at high risk for the progression of PTOA and could be applied for planning of treatment and rehabilitation strategies^35^.

## Methods

### Bovine articular cartilage harvest

Articular cartilage explants were harvested from the patellofemoral grooves of 1–2 week-old calves, obtained on the day of slaughter (Research ´87, Boylston, MA, USA). Full-thickness cartilage cylinders were prepared using a 3-mm dermal punch, and the top 1-mm disk (including the upper-most 100–200 µm of intact superficial zone) was harvested with a razor blade^36^. Cartilage disks were incubated in serum-free medium (low glucose Dulbecco’s Modified Eagles Medium [DMEM; 1 g/l]) supplemented with 10 mM HEPES buffer, 0.1 mM nonessential amino acids, 0.4 mM proline, 20 µg/ml ascorbate, 100 U/ml PenStrep, and 10% fetal bovine serum (FBS) and equilibrated for 2 days prior to the experiments (5% CO_2_; 37  C). During the experiments, the culture medium was changed every 2 days. Treatment groups were matched for location of the cartilage disks along the joint surface by distributing one disk from each region on the joint surface to each of the different treatment groups^5,37^.

### Injurious compression experiments

**Figure 1:**
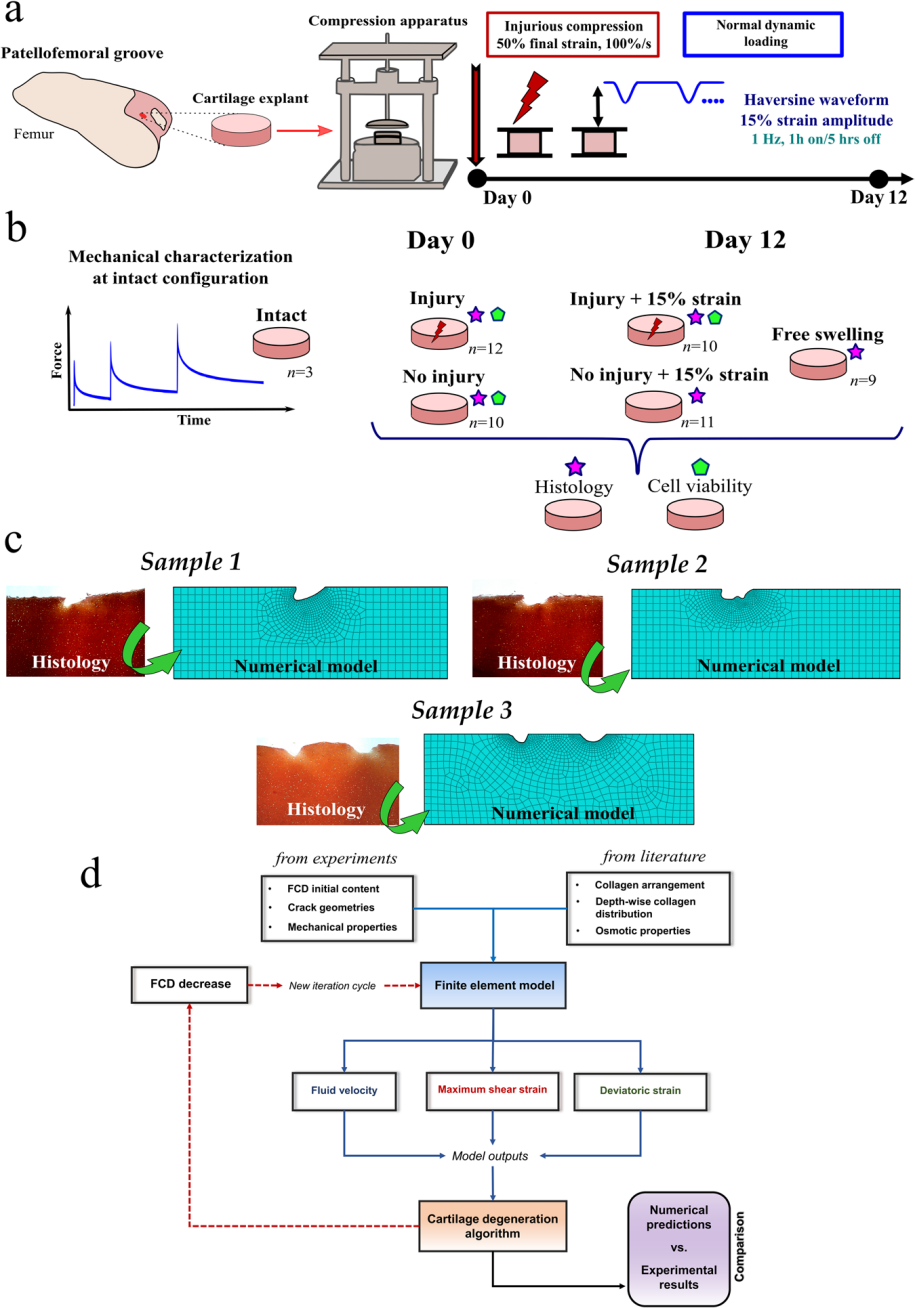
(**a**) Schematic of a custom-designed apparatus and timeline used to perform injurious dynamic compression experiments. Measurements at day 0 and day 12 were made for separate groups of cartilage explants. (**b**) Schematic of experimental design and number of samples in each group analyzed in the study. Groups with stars and polygons indicate histology and cell viability analysis, respectively. (**c**) Representative histological images with cartilage lesions, which were segmented to create finite element geometries. (**d**) General workflow of the iterative algorithm used to evaluate degeneration mechanisms. FCD initial content, sample geometries and mechanical properties were obtained from experimental measurements; collagen arrangement, depth-wise collagen distribution and osmotic properties were selected from the literature (See Table 1). Following a free-swelling step, injured explants were subjected to the dynamic loading protocol (unconfined compression, 15% strain amplitude, 1Hz). After mechanical simulation, fluid velocity, maximum shear strain and deviatoric strains were obtained and used for driving the cartilage softening algorithm, and new FCD content was implemented to the finite element model. This loop was repeated 50 times, and the FCD predictions were contrasted with the FCD decrease obtained from digital densitometry measurements.

Following pre-culturing for 2 days, on day 0, twenty two cartilage explants were injuriously compressed using a custom-designed, incubator-housed loading apparatus^5,38^. As previously described in detail^12^, each cartilage disk was subjected to unconfined compression at a strain rate of 100%/s to 50% final strain, followed by an immediate release at the same rate (Fig. 1a). Twelve cartilage explants from those twenty two were analyzed at day 0 and the remaining samples (*n* = 10) followed the dynamic protocol (see below). For all injured samples, we recorded in total 37 lesions, approximately on average 2 cracks per sample. All of the samples exhibited lesions after injurious compression.

### Dynamic loading compression experiments

Following the injurious compression on day 0, cartilage disks were placed in the 12-well polysulfone loading chamber with 0.3 ml culture medium and then subjected to dynamic compression. Cartilage disks were statically compressed to ensure a light contact, and unconfined dynamic compression (haversine waveform with 15% amplitude, mimicking “walking”)^5^ was then superimposed using a displacement-controlled loading (1 Hz) continuously for 1 h, followed by a 5 h rest period with the applied load removed (Fig. 1a). This dynamic loading cycle was repeated 4 times per day for 12 days. The control group (no injury + 15% strain) went through the same protocol, except that the initial injurious loading was not applied. Thus, dynamic compression at 15% strain amplitude was applied to two different groups of bovine cartilage explants simultaneously: **1)** 10 disks from injurious loading followed by 15% dynamic strain, **2)** 11 disks from no injurious loading followed by 15% dynamic strain. An additional 19 samples were immersed in the same culture medium but no dynamic loading was applied; 10 of those samples were analyzed at day 0 (no injury group) and nine samples at day 12 (free swelling group), respectively. To be consistent, the culture medium for all groups was changed every 2 days. A general schematic of experimental design is shown in Fig. 1b.

### Determination of biomechanical properties

To determine the mechanical parameters for the numerical models at the initial configuration, three additional intact bovine cartilage disks were placed between two impermeable platens in the polysulfone loading chamber and individually compressed in the incubator-housed loading apparatus. The thickness of each disk in the initial state of uniaxial, unconfined compression was quantified individually by applying a slow compression ramp (20 µm/s) with automated interrupt caused by an increase in the offset load, denoting a contact between the platen and the sample^36^.

To characterize the mechanical properties of these bovine cartilage disks, a pre-strain of 10% of the cartilage thickness was applied first (200 s compression, followed by 600 s relaxation). Then two additional compression-stress-relaxation steps of 2.5% strain, each, were applied (30 s compression, 300 s relaxation). Analysis of the mechanical testing data is described below.

### Depth-wise fixed charge density distributions

After the dynamic loading measurements, all samples were fixed in 10% formalin neutral buffer. Further, the samples were processed in graded alcohol solutions and embedded in paraffin. Histological sections were then cut perpendicular to the cartilage surface. Digital densitometry was used for quantifying the depth-wise optical density profiles^39^ for the five groups (Fig. 1b). Microscopic sections (thickness = 3 µm) were stained with Safranin-O in standardized conditions (pH 4.6). The amount of Safranin-O stain in the histological sections was determined by capturing grayscale images from the histological sections with a light microscope (Olympus CH-2, Olympus, Tokyo, Japan) and CCD camera (PerkinElmer, MA, USA). The grayscale images were converted to optical density (absorbance) maps by calibrating the system with a combination of five neutral density filters corresponding optical density values 1, 1.3, 1.6, 2 and 2.6 (Schott, Mainz, Germany). Three optical density image maps from three separate sections of each sample were analyzed and averaged perpendicular to the depth-wise direction to create a depth-wise profile of the optical density. The profiles were always measured in the mid-point between the lesion and the edge of the samples. This distance was not always the same in micrometers since the lesion location changed from sample to sample. Finally, based on the previous reports, the depth-wise optical density profiles were used for estimating the depth-wise distribution of the FCD content^40–42^.

### Cell viability experiments

To further study the effect of dynamic loading on local chondrocyte viability, experiments were performed to assess cell viability at day 0 and 12 days following the injury. 1 mm-thick slices were collected from 1–2 additional disks in each group using a scalpel and rinsed twice with PBS to remove media. Then, a viability assay solution (1 mL PBS, 2 µl fluorescein diacetate (FDA), 5 µl propidium iodide (PI)) was pipetted onto the cut slices in the Petri dishes and covered with aluminum foil for 15 minutes. Next, the slices were rinsed twice with PBS to remove excess stain and placed on microscope slides. Finally, the samples were assayed and imaged with two fluorescence microscope filters to produce an image for viability analysis. Fluorescein diacetate was used for staining viable cells green while propidium iodide (both from Sigma-Aldrich, St. Louis, MO, USA) stained dead cells red^5,43^.

### Mechanobiological model

2D finite element (FE) models were constructed for three representative samples with cracks observed after injurious compression experiments. Histological samples were imaged using a light microscope (Axioimager 2, Zeiss, Germany) and then the images were imported into Mimics v19.1 (Materialise, Belgium) where the cartilage lesions were manually segmented. The segmented geometries were imported and meshed with linear axisymmetric pore pressure continuum elements (type CPE4P) using Abaqus (v6.14, Dassault Systèmes Simulia Corp., Providence, USA; Fig. 1c). Mesh convergence was assured. Cartilage was modeled using a fibril-reinforced porohyperelastic material with Donnan osmotic swelling and chemical expansion^44^. Specifically, this constitutive model considers that the solid matrix is divided into a porous non-fibrillar part, describing mainly the proteoglycan matrix, and an elastic fibrillar network, representing the collagen fibrils, and the contribution of swelling caused by FCD. The total stress in the tissue is given by

1 $${{\boldsymbol{\sigma }}}_{{\rm{tot}}}={{\boldsymbol{\sigma }}}_{{\rm{s}}}+{{\boldsymbol{\sigma }}}_{{\rm{fl}}}={{\boldsymbol{\sigma }}}_{{\rm{f}}}+{{\boldsymbol{\sigma }}}_{{\rm{nf}}}-p{\bf{I}}-{T}_{{\rm{c}}}{\bf{I}}={{\boldsymbol{\sigma }}}_{{\rm{f}}}+{{\boldsymbol{\sigma }}}_{{\rm{nf}}}-{\rm{\Delta }}\pi {\bf{I}}-{\mu }^{{\rm{f}}}{\bf{I}}-{T}_{{\rm{c}}}{\bf{I}},$$

where ***σ***_tot_ is the total stress tensor, ***σ***_s_ and ***σ***_fl_ are the stress tensors of the solid and fluid matrices, respectively, *p* and ${\rm{\Delta }}{\rm{\pi }}$ are the hydrostatic and swelling pressure gradients, respectively, **I** is the unit tensor, *T*_c_ is the chemical expansion stress, *μ*^f^ is the chemical potential of water, and ***σ***_f_ and ***σ***_nf_ are the stress tensors of the fibrillar and non-fibrillar matrices, respectively. The non-fibrillar component is defined using a neo-Hookean material, in which the stress tensor is given by

2 $${{\boldsymbol{\sigma }}}_{{\rm{nf}}}={K}_{{\rm{nf}}}\frac{\mathrm{ln}(J)}{J}{\bf{I}}+\frac{{G}_{{\rm{nf}}}}{J}({\bf{F}}\cdot {{\bf{F}}}^{{\rm{T}}}-{J}^{\frac{2}{3}}{\bf{I}}),$$

where *K*_nf_ and *G*_nf_ are the bulk and the shear moduli of the non-fibrillar matrix, *J* is the determinant of the deformation gradient tensor **F**. The bulk (*K*_nf_) and shear (*G*_nf_) modulus are defined as

3 $${K}_{{\rm{nf}}}=\frac{{E}_{{\rm{nf}}}}{3(1-2{\nu }_{{\rm{nf}}})},$$

4 $${G}_{{\rm{nf}}}=\frac{{E}_{{\rm{nf}}}}{2(1+{\nu }_{{\rm{nf}}})},$$

where *E*_nf_ and *ν*_nf_ are the Young’s modulus and the Poisson’s ratio of the non-fibrillar matrix. The stress of a single collagen fibril *σ*_f_ is given by

5 $${\sigma }_{{\rm{f}}}=\{\begin{array}{cc}{E}_{{\rm{f}}}{\varepsilon }_{{\rm{f}}}, & {\varepsilon }_{{\rm{f}}} > 0\\ 0, & {\varepsilon }_{{\rm{f}}}\le 0\end{array},$$

where *E*_f_ is the fibril network modulus and *ε*_f_ is the fibril strain. Thus, the collagen fibrils resist only tension. The fibril network stress arises from the sum of primary and secondary collagen fibril stresses, which is calculated separately for each integration point in each element^44^. Stresses for these fibrils in tension were

6 $$\{\begin{array}{l}{\sigma }_{{\rm{f}},{\rm{p}}}={\rho }_{z}C{\sigma }_{{\rm{f}}}\\ {\sigma }_{{\rm{f}},{\rm{s}}}={\rho }_{z}{\sigma }_{{\rm{f}}}\end{array},$$

where *σ*_f,p_ and *σ*_f,s_ are the fibril stresses for primary and secondary fibrils, respectively, *C* is the density ratio between primary and secondary fibrils and *ρ*_*z*_ is the relative collagen density. Now, the stress tensor of the fibrillar matrix can be written as

7 $${{\boldsymbol{\sigma }}}_{{\rm{f}}}=\sum _{i=1}^{totf}{\sigma }_{{\rm{f}},i}{\overrightarrow{e}}_{{\rm{f}}}\otimes {\overrightarrow{e}}_{{\rm{f}}},$$

where *totf* is the total number of fibrils, *σ*_f,*i*_ is the stress of primary and secondary fibrils, ${\overrightarrow{e}}_{{\rm{f}}}$ is the collagen fibril orientation vector and ⊗ denotes outer product. Additionally, the Donnan osmotic swelling pressure at equilibrium can be determined as

8 $${\rm{\Delta }}\pi ={\varphi }_{{\rm{int}}}RT(\sqrt{{c}_{{\rm{FCD}}}^{2}+4\frac{{({\gamma }_{{\rm{ext}}}^{\pm })}^{2}}{{({\gamma }_{{\rm{int}}}^{\pm })}^{2}}{c}_{{\rm{ext}}}^{2}})-2{\varphi }_{{\rm{ext}}}RT{c}_{{\rm{ext}}},$$

where *ϕ*_int_ *ϕ*_ext_ ${\gamma }_{{\rm{int}}}^{\pm }$ and ${\gamma }_{{\rm{ext}}}^{\pm }$ are internal and external osmotic coefficients and internal and external activity coefficients, respectively, *c*_FCD_ is the fixed charge density content at equilibrium, *c*_ext_ is the external salt concentration (0.15 M), *R* is the molar gas constant (8.3145 J/mol K), *T* is the absolute temperature (293 K). The chemical expansion stress can be expressed as

9 $${T}_{{\rm{c}}}={a}_{0}{c}_{{\rm{FCD}}}\,\exp (-\kappa \frac{{\gamma }_{{\rm{ext}}}^{\pm }}{{\gamma }_{{\rm{int}}}^{\pm }}\sqrt{{c}^{-}\,({c}^{-}+{c}_{{\rm{FCD}}})}),$$

where *a*_0_ and *κ* are material constants^44^ and *c*^−^ is the mobile anion concentration. Finally, the fluid flow in the non-fibrillar matrix was modeled according to Darcy’s law:

10 $$q=-\,k\nabla p,$$

where *q* is the flow rate in the non-fibrillar matrix, *k* is the hydraulic permeability and ∇*p* is the pressure gradient vector across the region. The fluid velocity which travels through the porous medium can be determined as

11 $${v}_{{\rm{fl}}}=q(\frac{e+1}{e})=-\,k\nabla p(\frac{e+1}{e}),$$

where *v*_fl_ is the interstitial fluid velocity. The void ratio *e* of the material is the ratio of the fluid to solid:

12 $$e=\frac{{n}_{{\rm{fl}}}}{{n}_{{\rm{s}}}},$$

where *n*_s_ is the solid volume fraction and *n*_fl_ is the fluid volume fraction.

The depth-dependent collagen density content and orientation of cartilage, as implemented into the model, were obtained from experimental studies of bovine calves^36,45,46^ while the initial depth-wise distribution profile of the FCD was obtained as an average of the estimated optical density profiles of 12 samples that were analyzed by digital densitometry right after the injury. In order to obtain the FCD distribution, a sixth-degree polynomial function was fitted (with generalized least-squares) to the experimental depth-wise optical density profiles using Matlab (The MathWorks, Natick, MA, USA). Next, the depth-wise optical density profile was converted into the FCD profile by using the average value of the FCD (in mEq/ml) reported for calf femoral cartilage^47^. That is, the mean of the FCD profile calculated through tissue depth was the same as the mean of the FCD reported by Han *et al*.^47^. In addition, the biomechanical parameters (*E*_f_, *E*_nf_, and *k*) were obtained by fitting the numerical models to the unconfined compression experiments using Matlab by minimizing the normalized mean squared error between experimental and numerical forces (*fminsearch* function)^48^. A detailed list of the material parameters used in the models is given in Table 1.

**Table 1:** Material parameters for the fibril-reinforced porohyperelastic swelling cartilage model.

Material parameter	Value	Description
*E*_f_ (MPa)	20*	Fibril network modulus
*E*_nf_ (MPa)	0.16*	Nonfibrillar matrix modulus
*k* (10^−15^ m^4^/(Ns))	1.3*	Hydraulic permeability
*ν*_nf_ (−)	0.42**	Poisson’s ratio of the nonfibrillar matrix
*C*	3.009***	Ratio of primary to secondary collagen fibers
**Composition**		
*n*_fl_ (−)	0.85 − 0.15h***	Depth-wise fluid fraction distribution
*c*_FCD_ (mEq/ml)	−4.4*h*^6^ + 15.2*h*^5^ − 21.0*h*^4^ + 14.9*h*^3^ − 5.8*h*^2^ + 1.1*h* + 0.03^†^	Depth-wise fixed charge density distribution
*ρ*_z_ (−)	20.6*h*^6^ − 64.4*h*^5^ + 78.1*h*^4^ − 45.9*h*^3^ + 13.4*h*^2^ − 1.6*h* + 0.96^††^	Depth-wise collagen distribution

*Obtained by fitting the model to unconfined compression experiments.

**Obtained from Li *et al*.^74^, the effective Poisson’s ratio of the tissue became close to 0.1 due to reinforcement by the collagen fibrils.

***Obtained from Wilson *et al*.^44^.

^†^Obtained from optical density distributions and converted into FCD values by using the average value of FCD reported by Han *et al*.^47^.

^††^Obtained from Saarakkala and Julkunen^41^.

*h* indicates normalized distance from the cartilage surface (surface = 0, bottom = 1).

The following boundary conditions were applied to the mechanobiological models: the bottom of the explant was fixed in the axial direction and fluid flow was allowed through free and lesion surfaces. This fluid flow boundary condition was the same as used earlier^23,30^. The contact between the plate and cartilage was assumed impermeable and frictionless. Following an initial free-swelling step, the modeled explant was subjected to the unconfined dynamic compression protocol described in an earlier experimental section of this study.

### Cartilage degeneration algorithm

An iterative cartilage degeneration algorithm was developed to be dependent on three different model outputs: interstitial fluid velocity, maximum shear and deviatoric strains using Abaqus and Matlab. These outputs were passed to the algorithm, which assumed FCD loss to occur if either deviatoric strain (*ε*_dev_) exceeds a threshold of 20%, or maximum shear strain (*ε*_shr_) is more than 50% or fluid velocity (*v*_fl_) is greater than 0.04 mm/s during the entire loading cycle. The deviatoric strain can be defined by

13 $${\varepsilon }_{{\rm{dev}}}=\frac{1}{3}(\sqrt{{({\varepsilon }_{1}-{\varepsilon }_{2})}^{2}+{({\varepsilon }_{1}-{\varepsilon }_{3})}^{2}+{({\varepsilon }_{2}-{\varepsilon }_{3})}^{2}}),$$

where *ε*_1_, *ε*_2_ and *ε*_3_ are the principal strains. Therefore, the maximum shear strain is given by

14 $${\varepsilon }_{{\rm{shr}}}={\varepsilon }_{{\rm{\max }}}-{\varepsilon }_{{\rm{\min }}},$$

where *ε*_max_ and *ε*_min_ are the maximum and minimum principal strains, respectively. Following each degeneration simulation, the new FCD content was implemented into the finite element model and a new iteration cycle was started. The cartilage degeneration algorithm in combination with the finite element analysis was simulated for 50 iterative adaptive steps. That is, the algorithm was allowed to run sufficiently long (50 iterations) to guarantee that the equilibrium condition was reached.

The criteria regarding strains were based on earlier studies^15,17,49,50^ and an additional sensitivity analysis (strain threshold range was set from 0.15 to 0.7) seeking to adjust the connection between predictions and observations (data not shown). The fluid velocity threshold (0.04 mm/s) was defined based on preliminary tests where the initial fluid velocity value was obtained from the intact cartilage model (average in the explant at 15% strain of the dynamic loading protocol). Furthermore, a parametric analysis was conducted where the fluid velocity threshold was varied (range was set from 0.01 to 0.8 mm/s). This sensitivity analysis showed that for lower values of strain and fluid velocity, cartilage degeneration increases more rapidly and even in different areas than around the cracks (cartilage surface and sample edges). Clearly, these results were not in good agreement with those observed experimentally. In contrast, when we studied higher values for every threshold parameter, the degeneration algorithm revealed either a prolonged process to predict some FCD loss or even negligible degradation in the sample. Additionally, we tested different linear and nonlinear approaches to simulate the time-dependent evolution of the cartilage degradation. Each of them produced similar final results. Based on these tests, we decided to implement a piece-wise constant degeneration rate factor *D*_r_ defined as

15 $${D}_{{\rm{r}}}=\{\begin{array}{ll}0 & {\rm{if}}\,{\varepsilon }_{\beta } < {\varepsilon }_{\beta ,{\rm{thres}}},\\ 0.6 & {\rm{if}}\,{\varepsilon }_{\beta ,{\rm{thres}}}\le {\varepsilon }_{\beta }\le {\varepsilon }_{\beta ,{\rm{failure}}},\,\beta ={\rm{dev}},\,{\rm{shr}}\\ 0 & {\rm{if}}\,{\varepsilon }_{\beta }\le {\varepsilon }_{\beta ,{\rm{failure}}},\end{array}$$

where *ε*_*β*_ is the strain variable (either deviatoric (*β* = dev) or maximum shear strain (*β* = shr)), *ε*_*β*,thres_ is the strain threshold at which the non-fibrillar matrix damage initiates and *ε*_*β*,failure_ is the value for a complete failure of the ground substance (*ε*_*β*,failure_ = 1). Likewise, similar approach was used in case of the fluid velocity driven degeneration

16 $${D}_{{\rm{r}}}=\{\begin{array}{ll}0 & {\rm{if}}\,{v}_{{\rm{fl}}} < {v}_{{\rm{fl}},{\rm{thres}}},\\ 0.6 & {\rm{if}}\,{v}_{{\rm{fl}},{\rm{thres}}}\le {v}_{{\rm{fl}}}\le {v}_{{\rm{fl}},{\rm{failure}}},\\ 0 & {\rm{if}}\,{v}_{{\rm{fl}}}\le {v}_{{\rm{fl}},{\rm{failure}}},\end{array}$$

where *v*_fl,thres_ and *v*_fl,failure_ are the fluid velocity values at which the non-fibrillar matrix damage initiates (*v*_fl,thres_ = 0.04 mm/s) and the entire failure occurs (*v*_fl,failure_ = 1.5 mm/s), respectively. This degeneration rate function produced the results in a reasonable time.

It should be noted that this particular approach is able to produce a distinguishable cartilage deterioration and shows an excellent agreement between the experimental measurements (the FCD decrease in the injured explants) and modeled cartilage degeneration (see the results). To verify our approach, we tested the proposed degeneration mechanisms, as well as the thresholds for each mechanism using an intact cartilage explant in our mechanobiological model. As a result, no changes in the FCD content were observed in the models with normal, healthy cartilage surface.

A general workflow of our numerical approach is shown in Fig. 1d.

### Numerical parametric analysis

**Figure 2:**
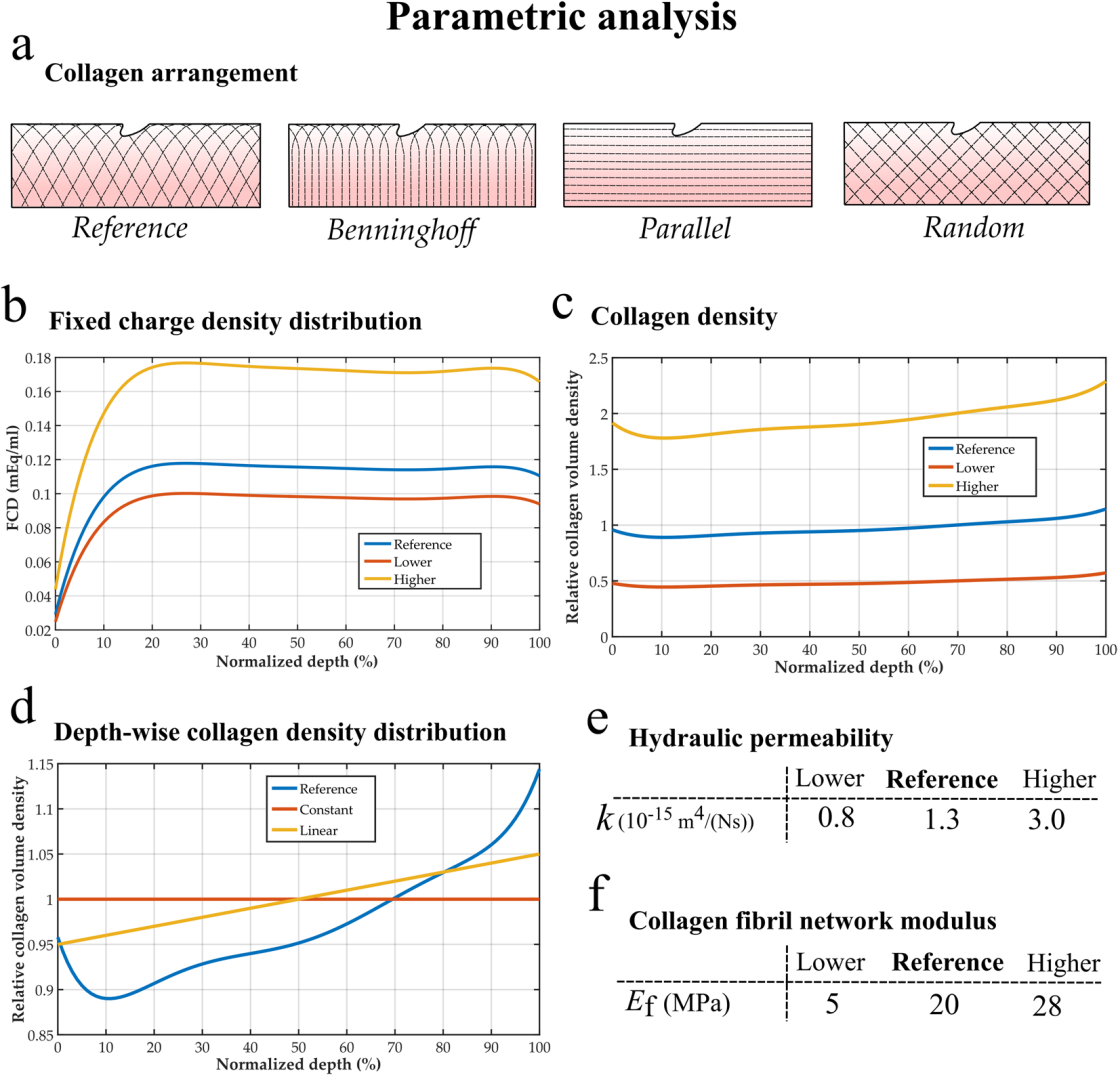
Parametric variation of tissue components in the mechanobiological model. (**a**) Collagen arrangements. (**b**) Initial fixed charge density distributions. (**c**) Relative collagen densities. (**d**) Depth-wise relative collagen density distributions. (**e**) Range of hydraulic permeability *k* and (**f**) collagen fibril network moduli *E*_*f*_.

Since the specific collagen fiber orientation and collagen density were not measured, the mechanobiological model was used for evaluating their influence on the FCD decrease predictions using one model (Sample 1). Furthermore, the initial FCD content, the permeability and the collagen fibril network modulus *E*_f_ were also varied in this analysis. Finally, in these parametric studies, we analyzed two main mechanisms of the cartilage degeneration based on preliminary results: fluid velocity and deviatoric strain. A complete illustration of these properties and their ranges used in the parametric study is given in Fig. 2.

### Statistical analysis

Anderson-Darling normality test was used to determine normality of the FCD content results. In addition, one-way ANOVA was conducted to determine statistical differences in the FCD content between the groups at both time points: injury and no-injury (control) at day 0; injury + 15% strain, no-injury + 15% strain, and free swelling at day 12. Statistical analyses were carried out using Matlab (Statistics and Machine Learning Toolbox^TM^) and the level of significance was set at *α* = 0.05.

## Results

### Fixed charge density content

**Figure 3:**
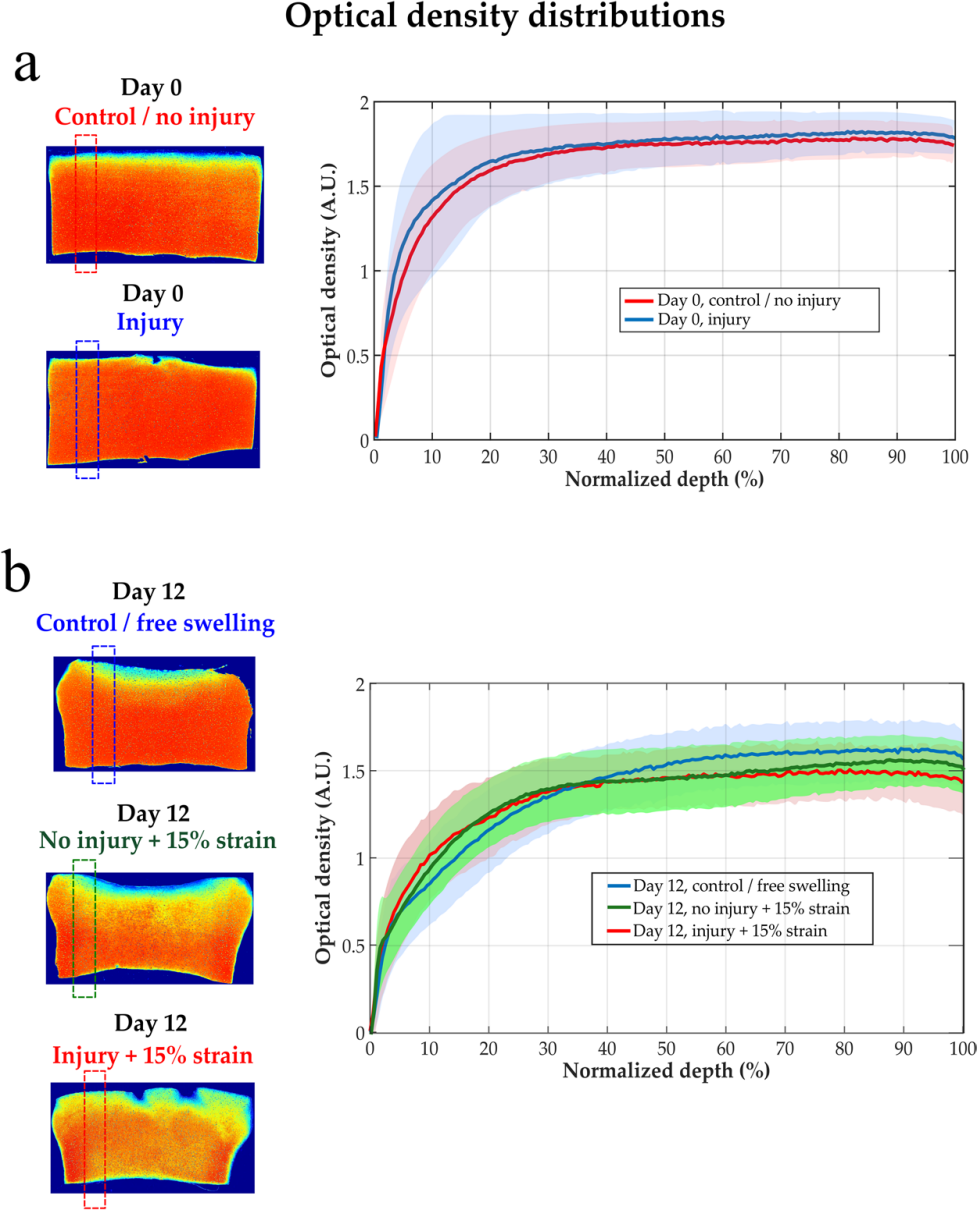
Optical density distributions (indicative of FCD content^40^) in (**a**) injury and control/no injury groups at day 0, and in (**b**) free swelling, no injury + 15% strain, and injury + 15% strain groups at day 12. There were no statistically significant differences between the evaluated groups when analyzed further away from the lesions (*p*> 0.05).

**Figure 4:**
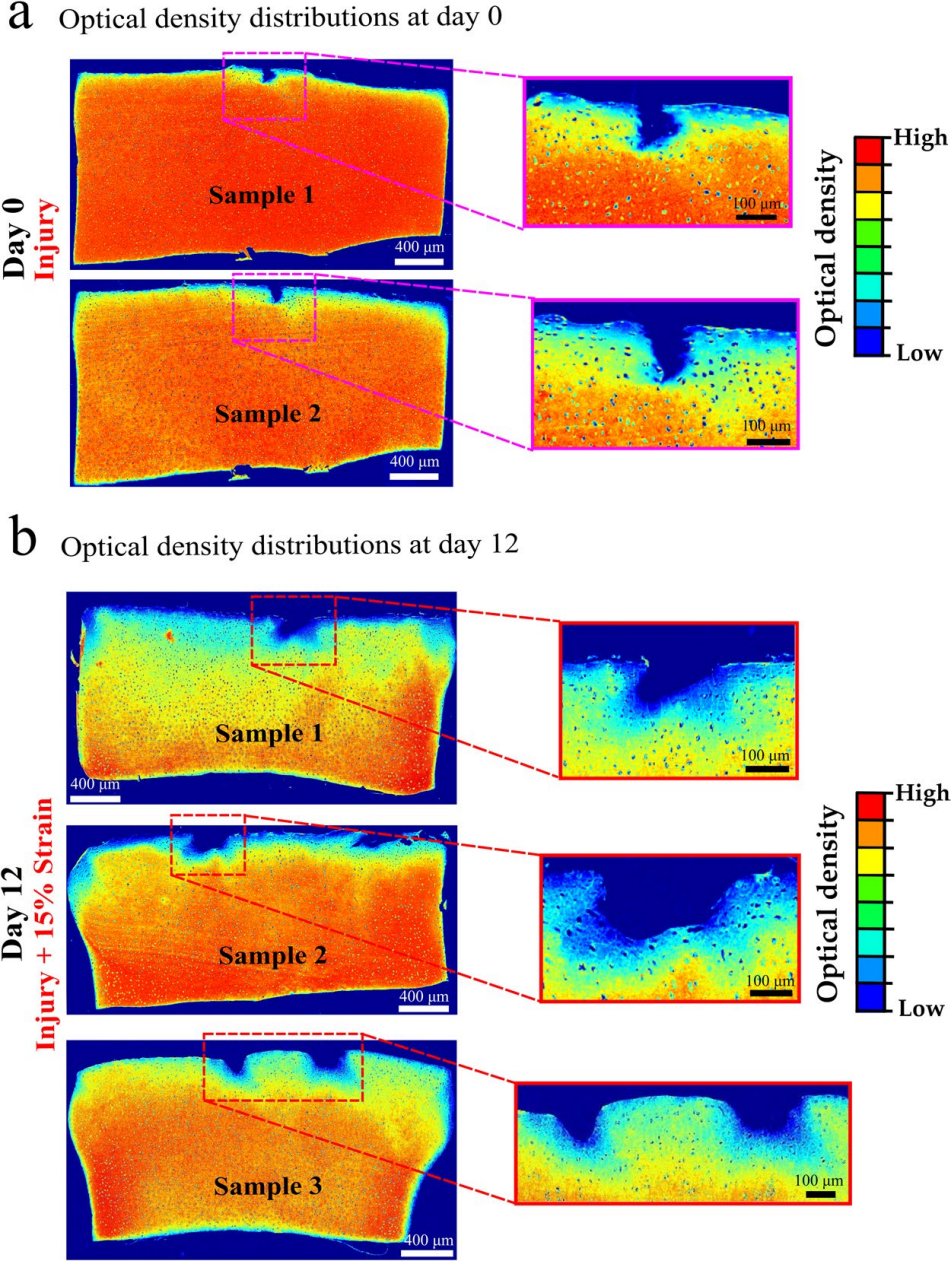
Representative optical density distributions, indicative of FCD content^40^, from independent samples in (**a**) injury samples at day 0 and (**b**) injury + 15% strain samples at day 12. Only small FCD loss was observed right after the injurious compression at day 0 while the localized FCD decrease was higher around tissue lesions at day 12.

The FCD content was similar in the injury and control groups along the tissue depth, when the measurements were conducted at certain distance from the cracks at day 0 (*p* = 0.505) (Fig. 3a). Similarly, after 12 days, the FCD content yielded no significant differences among the free swelling, no-injury and injury group (*p* = 0.861) through the sample depth when the analysis was performed further away from the lesions (Fig. 3b,c). Likewise, only negligible FCD loss was observed around lesions right after the injury at day 0. However, the cartilage explants with cracks at day 12 showed localized FCD decrease around the lesions and upper corners (Fig. 4).

### Chondrocyte viability analysis

**Figure 5:**
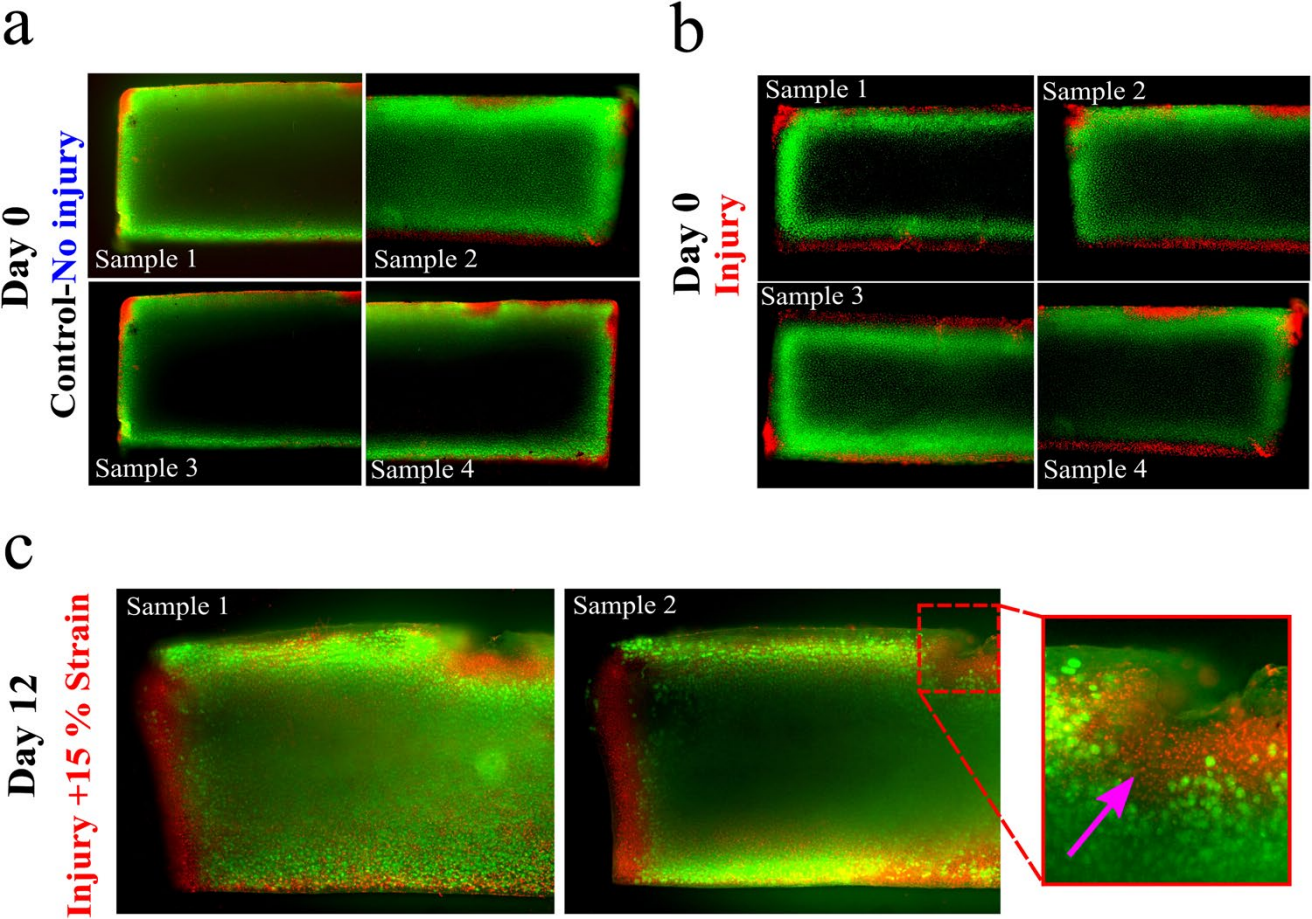
Fluorescently stained bovine samples to evaluate cellular viability after treatments for day 0 and day 12. Fluorescein diacetate was used to stain viable cells green while propidium iodide stained dead cells red. (**a**) Injury and (**b**) control/no injury groups at day 0 with minor cell death. (**c**) Injury + 15% strain group with localized chondrocyte death in the vicinity of the cracks (arrow).

Changes in cellular viability were noted in the cartilage explants with cracks after 12 days (Fig. 5). Extensive cell death was observed particularly around the lesions. Other areas further away from the cracks showed qualitatively similar cell viability as observed in the injury group at day 0 and in the control group.

### Computational degeneration predictions

**Figure 6:**
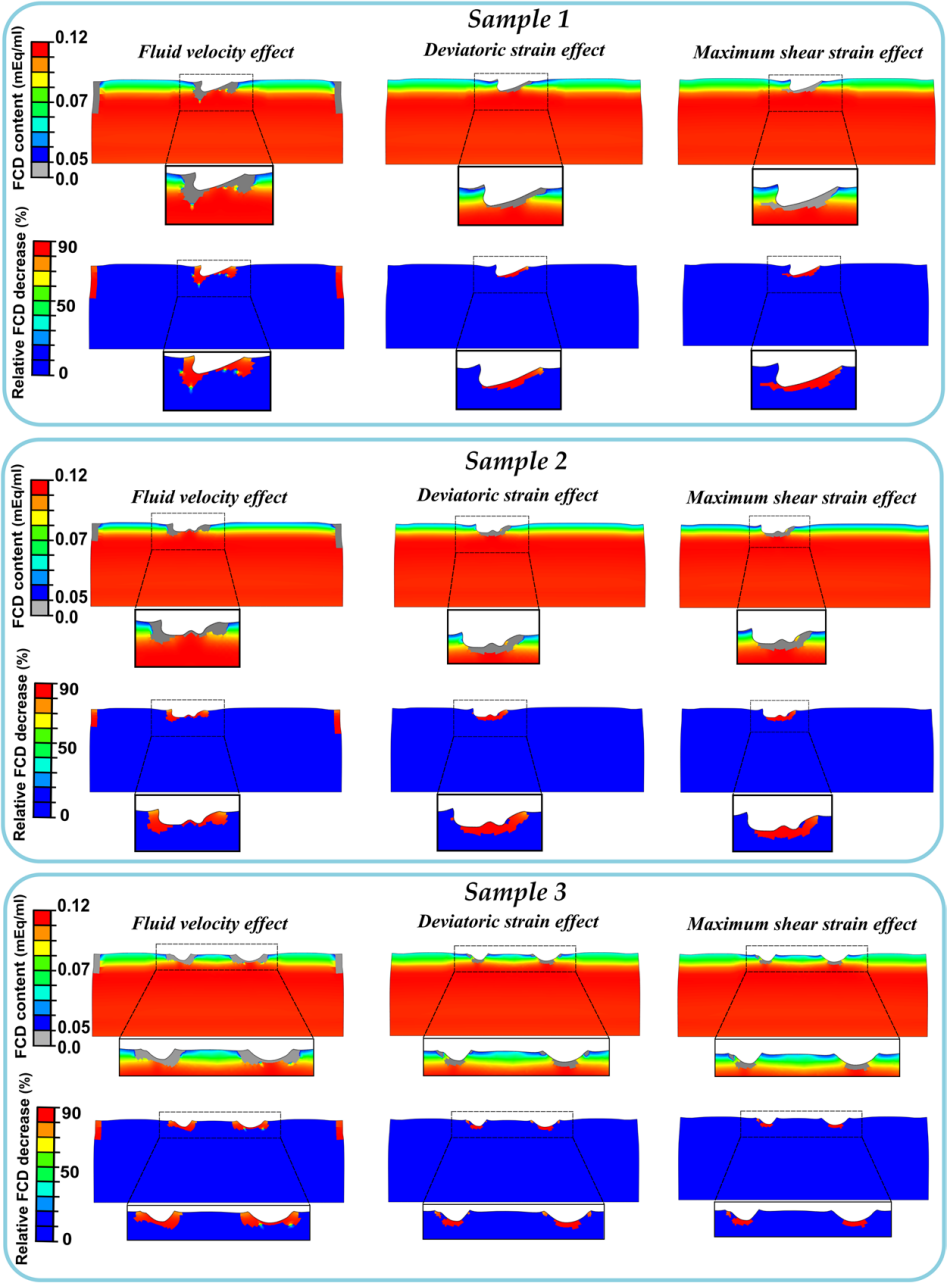
FCD content and relative FCD decrease distributions in the three different samples predicted by the models with fluid flow, deviatoric strain and maximum shear strain driven adaptive mechanisms. FCD content reduced in the upper corners and around the cracks when the degeneration algorithm was driven by the fluid velocity, while the deviatoric and maximum shear strain driven mechanisms showed slightly different and discontinuous FCD loss around the fissures.

**Figure 7:**
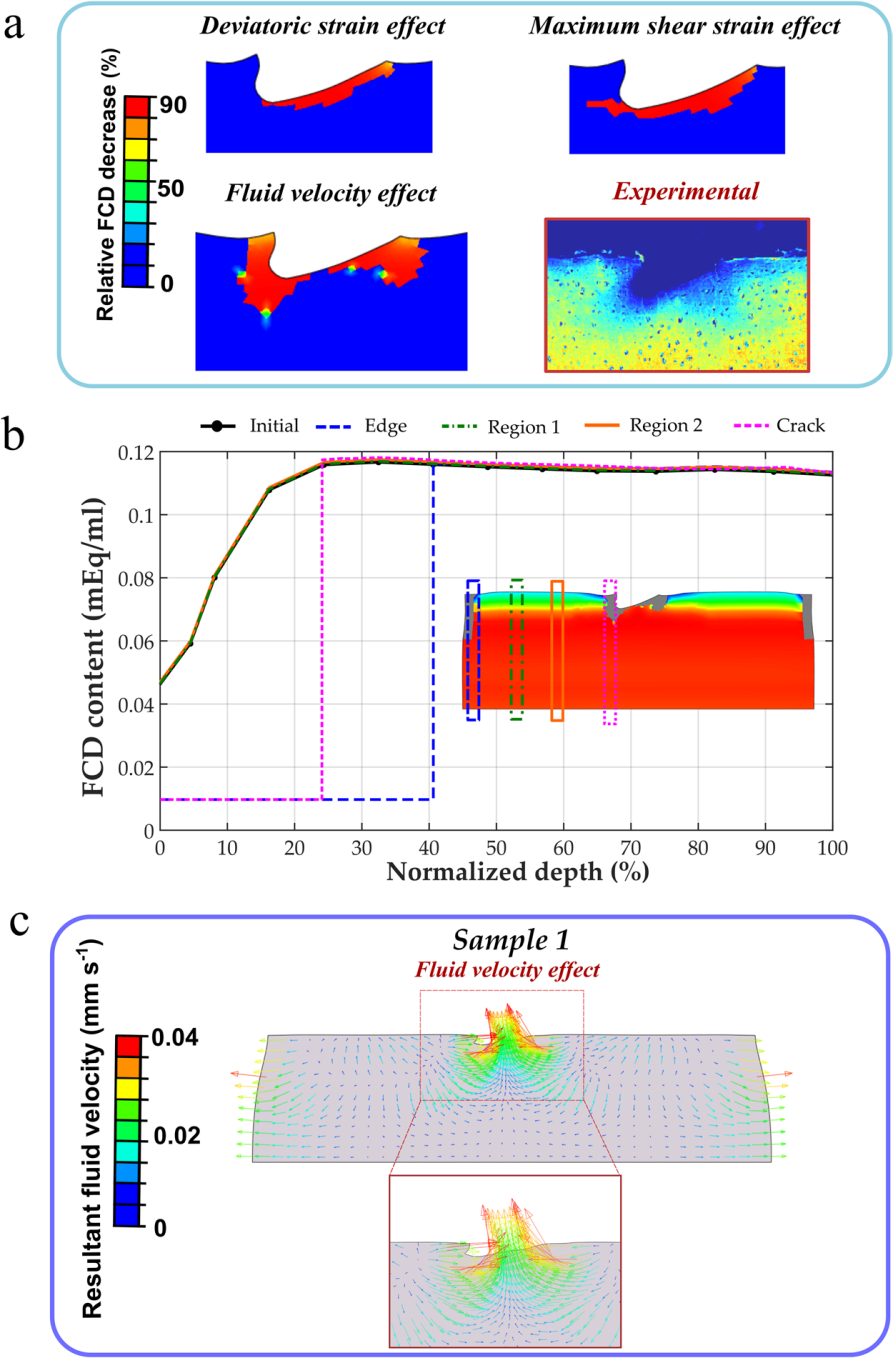
(**a**) Relative FCD decrease distributions obtained from the model with the fluid velocity degeneration mechanism exhibited a good agreement with the experimental measurements. (**b**) FCD content distributions, predicted by the model, along with the explant depth at different locations compared with the initial configuration (initial content overlapped with the region 1 and 2). Cartilage degeneration was dominant in regions close to the lateral edges and around the lesions. (**c**) Resultant fluid velocity vector in the cartilage explant at ~20% of the dynamic loading cycle. Remarkably fluid flow concentration was exhibited around the lesion.

The three representative samples showed that the FCD content decreased in the upper corners and in the vicinity of the lesions, when the degeneration algorithm was driven by the fluid velocity, while the deviatoric and maximum shear strain driven mechanisms exhibited a slightly different and discontinuous FCD loss around the cracks (Fig. 6). Specifically, the relative FCD decrease in the model with the fluid velocity effect illustrated a good correspondence with the experimental findings (Figs 6 and 7a). Main FCD decrease occurred in the regions close to the lateral edges and around the lesions, at depths of approximately 40% and 25% of the explant’s height, respectively (Fig. 7b). Notably, high fluid velocity was displayed around the crack at ~20% of the loading cycle, showing a large expulsion of fluid through the damaged surface (Fig. 7c).

#### Parametric analysis of the effect of tissue components

**Figure 8:**
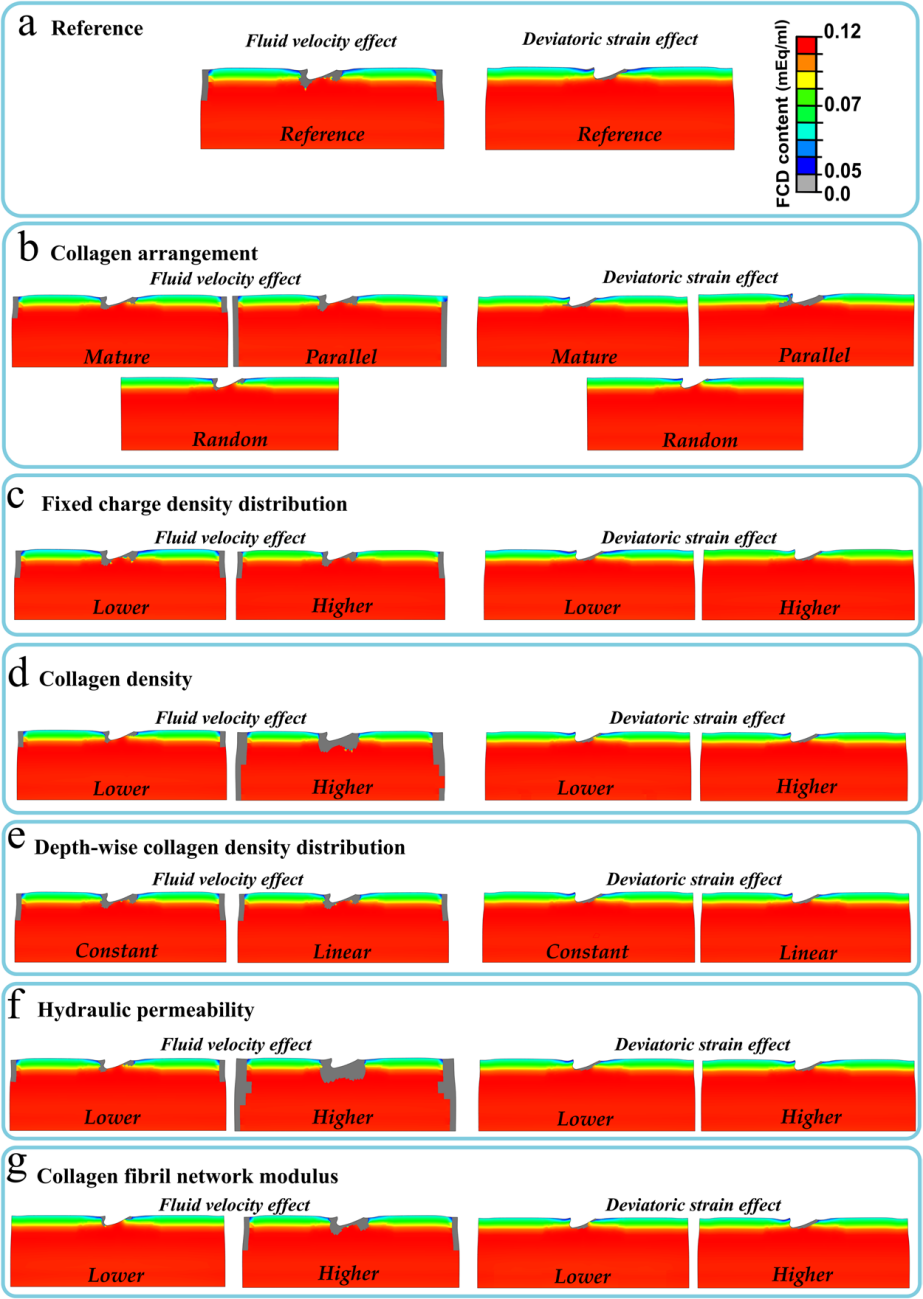
FCD content decrease distributions in the models with fluid velocity and deviatoric strain degenerative mechanisms in (**a**) reference explant model and after parametric variation of the tissue components: (**b**) collagen arrangement, (**c**) initial FCD content, (**d**) collagen density, (**e**) depth-wise collagen density distribution, (**f**) hydraulic permeability and (**g**) fibrillar network modulus (see Fig. 2).

The parametric analysis within the mechanobiological model by varying the collagen arrangement showed slightly higher FCD loss when the collagen fibrils were oriented parallel to the surface; a minimal effect was observed by the rest of the collagen arrangements (Fig. 8a vs b). Variations of the initial FCD in the model showed only small changes in the numerical FCD loss predictions (Fig. 8a vs. c). Increases in the collagen density exhibited a substantial influence on the FCD loss when the fluid velocity mechanism was utilized, however this effect was minimal with the deviatoric strain principle (Fig. 8a vs. d). In contrast, altering only the depth-wise collagen density distribution revealed only slight changes on the cartilage FCD (Fig. 8a vs. e).

On the other hand, an increase in the hydraulic permeability (*k*) revealed considerable FCD decrease associated with the fluid velocity mechanism; however, minimal influence was observed with the deviatoric strain mechanism (Fig. 8a vs. f). In addition, increase in the collagen fibril network modulus (*E*_f_) showed higher FCD loss with the fluid flow mechanism, while it did not affect substantially to the FCD loss with the deviatoric strain mechanism (Fig. 8a vs. g).

## Discussion

In the present study, we investigated how different mechanisms (fluid velocity, deviatoric and maximum shear strains) could cause PG loss in impact-injured articular cartilage under mechanical, physiologically relevant dynamic loading. For this, we employed a novel mechanobiological model accompanied with experimental findings. Our degeneration algorithm predictions agreed well with the experimentally observed FCD content and cell death, supporting the validity of our approach. Numerical results suggest that both the fluid velocity and deviatoric strain mechanisms could be plausible, though the fluid velocity mechanism seems to correspond better with the experimental findings. Therefore, we suggest that the FCD decrease following cartilage injury and subsequent tissue loading, in the absence of inflammatory challenge, might be caused simply by an easy leakage of the PGs through the damaged surface by high fluid outflow.

Digital densitometry measurements indicated that the FCD quantity (*i.e*. PG content) was not altered further away from the lesion during the 12 day measurement period. In contrast, the concentrated FCD decrease was revealed around the cracks during the cyclic loading after 12 days. Consistently, the cellular viability analysis also demonstrated that moderate dynamic loading caused chondrocyte death in the vicinity of the lesions. These alterations indicate that the disruption of the cartilage homeostasis is a location-specific and the injurious impact contributes to chondrocyte death and subsequently loss of PGs over time. These results are consistent with the findings reported in the literature, in which post-traumatic tissue evolution revealed a decrease in chondrocyte viability and PG content 7 or 14 days after injury^51–53^.

Parametric analysis via the mechanobiological model suggests that the collagen density, collagen network modulus and hydraulic permeability (which “controls” fluid velocity) are predominant parameters that affect cartilage FCD loss, especially in the model with fluid velocity-controlled tissue degeneration. In contrast, the effect of different collagen arrangements on the changes of the FCD content was small. Consistent with these findings, the depth-wise collagen density distribution had virtually no influence on prediction of decreases in FCD. Nonetheless, independent of the initial cartilage structure or material properties, we always ended up to the same conclusion in terms of the location of the FCD loss.

Similar approaches for iterative tissue adaptation as applied here have been used to study cartilage mechanics^24,54–57^, bone remodeling^29,58–62^ and tissue engineering characterization^63,64^. A critical factor in the model generation and simulation is the selection of the degeneration thresholds. The current values were estimated from earlier studies and after several additional simulations (sensitivity analysis, ranges given in the methods), contrasting between the numerical predictions and experimental FCD decrease observations. In agreement with our values, previous studies have reported thresholds^15,17,29^ and failure strains close to our values^13,14,16,50^. Likewise, other investigations have established similar fluid velocity values to predict bone formation and mass transport processes in cartilage^29,56,65^.

One might also speculate that knee joint trauma causes a loss of cartilage FCD in additional regions than just local to the mechanically-induced lesion due to cytokine-induced proteolytic degradation. However, in our study, the experiments were performed in the absence of additional exogenous cytokines. We found that in the absence, the FCD content was not changed significantly in the normal, healthy parts of the tissue further away from the lesions (when compared to the no-injury and free swelling groups at 12 days). In reality in the joint, a more widespread loss of FCD-associated aggrecan fragments could be linked to biochemical degradation and inhibition of normal aggrecan biosynthesis caused by the release of inflammatory cytokines^6,12^.

The FCD decrease around cartilage lesions was observed only after the 12-day dynamic loading was applied, and only negligible or small FCD loss was observed right after the injurious compression at day 0. This result is consistent with earlier studies of degeneration in young and adult cartilage^5,66^, suggesting that PG depletion and cell death in injured cartilage can be progressed by normal mechanical loading, not just by the injurious loading itself.

These promising results might open a possibility for this model to be employed to predict a subject-specific progression of PTOA and to evaluate the effect of clinical interventions in the future. Specifically, this model could show high and low risk lesions associated with PTOA and suggest an optimal and subject-specific rehabilitation protocol. However, assuming that the FCD loss following tissue injury and local collagen damage is directly caused by an easy escape of PGs through the lesion by fluid outflow, makes the prevention of the FCD loss highly problematic. In this case, potential treatment options should target methods that would be able to decrease the fluid outflow. This could be achieved with a restoration of native tissue’s collagen structure, proteoglycan content and/or permeability. On the other hand, if some part of the degeneration is driven by excessive tissue strains, then well-planned moderate, low amplitude^5^ joint loading and rehabilitation could delay further progression of PTOA.

Some limitations exist in this study regarding the experimental part, and model generation and assumptions. First, although young bovine cartilage might not capture all aspects of normal adult human cartilage behavior, it is much simpler to obtain normal, similarly aged (and therefore repeatable) intact joint cartilage, and a less expensive option for this proof-of-concept study. In addition, sensitivity analysis showed that different collagen arrangement and density, FCD content, and mechanical properties of cartilage changed the amount of tissue degeneration, but the locations of degeneration did not change. Some of those models could represent more mature human tissue. This analysis suggests that independent of the initial cartilage structural and mechanical properties, the conclusions would be the same. However, in future studies we will confirm the results by using adult human cartilage tissue. Second, small cell death at the sample edges was observed in both groups at day 0, which was probably caused by the sample preparation. However, this limitation does not change the conclusion that normal dynamic loading produces cell death around the cartilage lesions. Ongoing studies focus on the quantification of cell viability results and it will be part of a future investigation. Third, tissue crack geometries were segmented in a particular time and we did not consider their propagation over time. We also could not measure the relative change in a crack depth because the samples at 0 and 12 days were not the same. However, the predictions of the FCD decrease obtained using the realistic cartilage lesions at day 12 concurred well with the experimental findings. Crack propagation might be implemented through mesh-dependent damage evolution theory, enhanced gradients methods and accounting nonlinear effects of mechanical loading on a crack growth in the tissue^67–70^. However, experimental validation of this approach might be very challenging. Fourth, 3D continuum geometries rather than 2D may be required to consider the effect of complex discontinuities of 3D geometries as well as for patient-specific future implementations^30,71^. Also, collagen damage progression was not taken into account, because we assumed that the damage in the ground substance and the subsequent FCD loss appear before the disorganization of the collagen network, which has been observed previously, especially at a relatively short follow-up period^21^. Future efforts will be focused on combining the adaptation of the collagen content and fibril orientation^23,72^ as well as inflammatory responses^5,73^ with the studied degeneration mechanisms after a traumatic joint injury. For those, more experimental data are required.

In conclusion, our novel mechanobiological model was able to localize and predict changes in cartilage FCD content around lesions similarly with corresponding experiments. Both of the suggested mechanisms, fluid velocity or shear/deviatoric strain controlled, could be plausible to control cell death and/or tissue degradation, though the fluid velocity-controlled mechanism was in a better agreement with the distribution of the experimentally observed FCD loss. These findings and suggestions are relevant to consider in mechanobiological models to explore current and new potential treatment options associated with early cartilage damage that can progress to PTOA.

## Data Availability

The datasets generated during and/or analysed during the current study are available from the corresponding author on reasonable request.
